# Targeting BRD/BET proteins inhibits adaptive kinome upregulation and enhances the effects of BRAF/MEK inhibitors in melanoma

**DOI:** 10.1038/s41416-019-0724-y

**Published:** 2020-01-14

**Authors:** Manoela Tiago, Claudia Capparelli, Dan A. Erkes, Timothy J. Purwin, Shea A. Heilman, Adam C. Berger, Michael A. Davies, Andrew E. Aplin

**Affiliations:** 1 Department of Cancer Biology, Thomas Jefferson University, Philadelphia, PA 19107 USA; 2 Department of Surgery, Thomas Jefferson University, Philadelphia, PA 19107 USA; 3 Sidney Kimmel Cancer Center, Thomas Jefferson University, Philadelphia, PA 19107 USA; 4 Department of Melanoma Medical Oncology, The University of Texas MD Anderson Cancer Center, Houston, TX 77030 USA

**Keywords:** Melanoma, Tumour heterogeneity, Molecular medicine

## Abstract

**Background:**

BRAF-mutant melanoma patients respond to BRAF inhibitors and MEK inhibitors (BRAFi/MEKi), but drug-tolerant cells persist, which may seed disease progression. Adaptive activation of receptor tyrosine kinases (RTKs) has been associated with melanoma cell drug tolerance following targeted therapy. While co-targeting individual RTKs can enhance the efficacy of BRAFi/MEKi effects, it remains unclear how to broadly target multiple RTKs to achieve more durable tumour growth inhibition.

**Methods:**

The blockage of adaptive RTK responses by the new BET inhibitor (BETi), PLX51107, was measured by RPPA and Western blot. Melanoma growth was evaluated in vitro by colony assay and EdU staining, as well as in skin reconstructs, xenografts and PDX models following BRAFi, MEKi and/or PLX51107 treatment.

**Results:**

Treatment with PLX51107 limited BRAFi/MEKi upregulation of ErbB3 and PDGFR-β expression levels. Similar effects were observed following BRD2/4 depletion. In stage III melanoma patients, expression of BRD2/4 was strongly correlated with ErbB3. PLX51107 enhanced the effects of BRAFi/MEKi on inhibiting melanoma growth in vitro, in human skin reconstructs and in xenografts in vivo. Continuous triple drug combination treatment resulted in significant weight loss in mice, but intermittent BETi combined with continuous BRAFi/MEKi treatment was tolerable and improved durable tumour inhibition outcomes.

**Conclusions:**

Together, our data suggest that intermittent inhibition of BET proteins may improve the duration of responses following BRAFi/MEKi treatment in BRAF-mutant melanoma.

## Background

New therapeutic approaches have changed the standard-of-care for patients with v-Raf murine sarcoma viral oncogene homolog B (BRAF) V600-mutant melanoma.^1,2^ Immune checkpoint inhibitors often provide at least 3 years of relapse-free survival, particularly when a complete response (CR) is achieved.^3,4^ Targeted agents for BRAF and mitogen-activated protein kinase (MEK) elicit rapid responses in BRAF V600E/K-mutant melanoma patients as monotherapy and in combination.^5^ While these new therapies represent significant progress and 4-year overall survival is up to 53% with the combination of nivolumab plus ipilimumab,^6^ the 20% durable CR with immunotherapy leaves room for improvement, and many BRAF V600E/K melanoma patients treated with targeted inhibitors develop acquired resistance within ~1 year.^7,8^ In addition, some patients who achieved CR with BRAFi/MEKi therapy have experienced tumour recurrence following treatment cessation.^9,10^ Thus, optimisation of immune checkpoint and targeted inhibitor strategies with other therapeutics is important for maximising initial responses and achieving more durable effects.

A major mechanism that enables tumour cells to tolerate BRAF/MEK pathway inhibition is adaptive upregulation and/or activation of receptor tyrosine kinases (RTKs).^7,11^ Multiple RTKs, including v-erb-b2 avian erythroblastic leukaemia viral oncogene homolog 3/human epidermal receptor 3 (ErbB3/HER3), insulin-like growth factor 1 receptor (IGF-1R), platelet-derived growth factor receptor beta (PDGFR-β), tyrosine-protein kinase receptor UFO (AXL), and vascular endothelial growth factor receptor 2 (VEGFR-2), are upregulated and/or show enhanced activation following BRAFi/MEKi treatment.^11,12^ These adaptive responses are often influenced by the tumour microenvironment since the ligands for RTKs are highly expressed in stromal cells.^13^ RTK upregulation following BRAFi/MEKi treatment stimulates phosphatidylinositol-4,5-bisphosphate 3-kinase (PI3K) signalling that compensates for inhibition of the MEK–ERK1/2 pathway.^7,14^ Unfortunately, targeting PI3K in combination with MEK–ERK1/2 pathway inhibitors is challenging due to toxicity issues.^15^ While targeting ErbB3 enhances the response to BRAFi in BRAF-mutant melanoma models,^16^ other adaptive RTK responses likely arise. Thus, alternative approaches to broadly inhibit adaptive responses to BRAFi/MEKi in melanoma are needed.

Bromodomain and extra-terminal domain (BET) protein family, including BRD2, BRD3, and BRD4, regulates transcriptional programmes.^17,18^ BET proteins are epigenetic readers that bind to acetylated histones at promoters, enhancers, and super-enhancers,^19,20^ and regulate the expression of genes related to cell proliferation and apoptosis, such as *MYC* and *BCL2*.^17^ BET inhibitors (BETi), including JQ1 and OTX015, competitively displace BRD2/3/4 from acetylated histones, resulting in the suppression of BRD/BET-dependent genes in breast and ovarian cancer models.^21,22^ JQ1 also reduces the expression of RTKs and inhibits tumour cell growth and survival.^17,18^ Thus, BET inhibition may prevent RTK upregulation induced as an adaptive response to BRAF/MEK inhibition, and in doing so, enhances the effects of the targeted agents.^18,23,24^ While BETi have produced promising results in several malignancies, they have also been associated with toxicities and remain to be fully tested in BRAF-mutant melanoma.^25,26^

Here, we tested the role of BRD/BET proteins in the upregulation of RTKs and resistance to BRAFi/MEKi therapy in BRAF V600E melanoma models. We utilised a new BETi, PLX51107, and an intermittent schedule to improve the efficacy of the BRAFi/MEKi combination while minimising toxicity. We show that PLX51107 treatment reduces expression of ErbB3 and PDGFR-β. The effects were associated with BRD2 and BRD4 expression. PLX51107 enhanced the effects of BRAFi/MEKi on melanoma cell growth in monolayer cultures and in a three-dimensional (3D) human skin reconstruct model. Using an intermittent schedule, we showed that tumour reoccurrence following drug withdrawal was reduced in tumour-bearing mice treated with intermittent BETi with continuous BRAFi/MEKi compared with BRAFi/MEKi alone. Together, our data suggest that inhibition of BET proteins improves the efficacy of BRAFi/MEKi in BRAF-mutant melanoma.

## Methods

### Cell culture

A375 cells were cultured in Dulbecco's modified Eagle’s medium (DMEM, GIBCO, Life Technologies, Grand Island, NY, USA) supplemented with 10% foetal bovine serum (FBS, Life Technologies). M238 cells were cultured in RPMI 1640 medium (GIBCO) supplemented with 10% FBS and 2 mM L-glutamine (GIBCO). 1205Lu cells were cultured in MCDB 153 medium containing 20% Leibovitz-L15 medium, 2% FBS, 0.2% sodium bicarbonate, and 5 μg/mL insulin. All melanoma cell lines used in this study were validated as BRAF V600 mutants by Sanger sequencing. In addition, STR analysis was performed for the cell lines described above. Primary culture of normal keratinocytes (HFK), normal melanocytes (HFM), and normal fibroblasts (HFF) were isolated from human neonatal foreskin samples and cultured as previously described.^27^ All cells were grown with 1% penicillin/streptomycin (100 U/mL) added to all media at 37 °C in a humidified incubator supplemented with 5% CO_2_. Cells were routinely assayed for mycoplasma contamination with MycoScope Kit (Genlantis).

### Reverse-phase protein array analysis

Cells (2.0 × 10^5^/well) were seeded in six-well plates in normal growth media. The next day, cells were treated with either dimethyl sulfoxide (DMSO) or BRAFi/MEKi (dabrafenib, 50 nM + trametinib, 5 nM) ± increasing doses of the BETi JQ1 (0.1, 1, and 5 μM) or PLX51107 (1, 2, and 4 μM) for 24 h. Lysates from three independent experiments were processed and analysed, as previously described.^28^ Reverse-phase protein array (RPPA) data were used to determine antibodies that were significantly different between BRAFi/MEKi ± BETi-treated groups for each cell line. Comparisons were performed by the two-sample *t*-test method with 1000 permutations and assumed unequal variance. Antibodies with a *p*-value < 0.05 were considered significant. Statistical calculations were performed in Matlab® (v2015b) using the mattest function. Data points are shown as averages of three experimental replicates.

### siRNA transfections

BRD2 siRNA (GAAAAGAUAUUCCUACAGA), BRD4 siRNA (UGAGAAAUCUGCCAGUAAU), and negative control siRNA (siCONTROL Non-Targeting siRNA #1 UAGCGACUAAACACAUCAAUU) were purchased from Dharmacon Inc. (Lafayette, CO). Cells were transfected with chemically synthesised siRNAs at a final concentration of 20 nM using Lipofectamine RNAiMAX (Invitrogen, Carlsbad, CA).

### Western blot

Cells were washed twice in cold PBS and lysed with Laemmli sample buffer with β-mercaptoethanol. Proteins were resolved by SDS-PAGE and transferred to PVDF membranes. After blocking in 5% BSA, membranes were incubated with the indicated primary antibodies overnight at 4 °C, followed by incubation with peroxidase-coupled secondary antibodies. Immunoreactivity was detected using horseradish peroxidase (HRP)-conjugated secondary antibodies (CalBioTech, Spring Valley, CA) and chemiluminescence substrate (Thermo Fisher Scientific, Rockford, IL) on a Versadoc imaging system (Bio-Rad). The primary antibodies used were as follows: phospho-ErbB3 (Tyr-1197, #4561), ErbB3 (#4754), PDGFR-β (#3169), AXL (#4939), BRD2 (#5848), BRD4 (#13440), phospho-ERK1/2 (Thr-202/Tyr-204, #9101), ERK1/2 (#9102), and GAPDH (#2118) from Cell Signaling Technology (Danvers, MA); ERK2 (sc-1647) antibody was purchased from Santa Cruz Biotechnology Inc. (Dallas, TX).

### RTK cell surface expression

Cells (2.0 × 10^5^/well) were plated in six-well plates and treated, as indicated. At the end of the experiment, cells were detached using 2 mM EDTA and washed twice with cold PBS. Samples were stained with Zombie NIR (BioLegend, San Diego, CA), for 10 min at room temperature in the dark. Following two washes with FACS buffer (PBS with 1% FBS and 0.5% sodium azide), cells were stained with ErbB3-PE (catalogue #324705, 1:200, BioLegend) for 30 min at room temperature. Cells were then fixed with BD Cytofix/Cytoperm fixation/permeabilisation solution kit (BD Biosciences, San Jose, CA) and analysed on BD FACSCelesta Flow Cytometer (BD Biosciences) using FlowJo software (TreeStar, Ashland, OR). Data points are shown as averages of three experimental replicates.

### Cell growth assay

Cells (8 × 10^3^/well) were seeded in six-well plates. Cells were then treated with either DMSO or dabrafenib, 50 nM + trametinib, 5 nM ± JQ1 (0.1, 1, and 5 μM) or PLX51107 (1, 2, and 4 μM) three times per week, for 3 weeks, as indicated. Cells were then washed with PBS and stained with 0.2% crystal violet in 10% buffered formalin for 20 min. Subsequently, wells were washed and air-dried. Plates were scanned, and pictures were taken with a Nikon Eclipse Ti inverted microscope with NIS-Elements AR 3.00 software (Nikon, Melville, NY).

### S-phase entry analysis

Cells (2 × 10^5^/well) were seeded in six-well plates. Cells were then treated with the drug of interest for 48 h. The thymidine analogue EdU was added at a final concentration of 10 μmol/L for the final 16 h. EdU incorporation was measured using the Click-iT Plus EdU Alexa Fluor 647 Flow Cytometry Assay Kit (Thermo Fisher Scientific) and was utilised as per the manufacturer’s instructions. EdU staining was analysed on BD FACSCelesta Flow Cytometer (BD Biosciences) using FlowJo software (TreeStar, Ashland, OR). Data points are shown as averages of three experimental replicates.

### Growth factors and inhibitors

Recombinant human NRG1 was purchased from Cell Signaling Technology. Dabrafenib, trametinib, and JQ1 were purchased from Selleck Chemicals LLC (Houston, TX). PLX51107 was a gift from Plexxikon Inc. (Berkeley, CA).

### Kinetic growth assay

For real-time analysis of cell viability, cells (5 × 10^3^/well) were seeded in twelve-well plates. Phase-contrast images were taken every 2 h using an IncuCyte® live cell imager (Essen Biosciences, Ann Arbor, MI). Cells were treated three times a week of treatment of choice, and cell confluence of the cultures was measured using IncuCyte software over 3 weeks in culture.

### Human skin reconstructs containing melanoma cells

The skin reconstruct model was performed with minor changes from Sandri et al.^27^ A375 and 1205Lu melanoma cells were incorporated into the epidermis at the time of keratinocyte seeding (one melanoma cell to three keratinocytes). After 24 h of submersion, skin reconstructs were then transferred to the air–liquid interface and maintained for 2 weeks in culture medium composed of DMEM/Ham’s F-12 medium mixture (3:1) supplemented with 10% FBS, 0.1 nM cholera toxin, 5 μg/mL insulin, 5 μg/mL apo-transferrin, 0.4 μg/mL hydrocortisone-21, and 0.5 ng/mL epidermal growth factor (EGF).

### Skin reconstruct quality control and immunohistochemistry

After the keratinocyte cell stratification and melanoma invasion period (14 days), skin reconstructs were fixed with 10% buffered formalin at 4 °C and histologically processed for inclusion in paraffin. Sections of 5 μm were stained with haematoxylin/eosin (H&E) and analysed for their morphology. The quality of the skin reconstruct was considered satisfactory when a differentiated and stratified epidermis similar to human epidermis was observed. For immunohistochemical analysis, skin reconstructs were stained for pan-cytokeratin (catalogue #914204, 1:1000, BioLegend), and Ki-67 (catalogue #ab15580, 1:200, Abcam, Cambridge, UK). The photomicrographs were obtained by optical microscopy, using a Nikon Optiphot microscope (Shinagawa, Tokyo, Japan), and analysed using the NIS-Elements software (Nikon).

### Tumour xenograft experiments

For experiments involving 1205Lu and A375 cells, athymic mice (J:NU, Homozygous, The Jackson Laboratory, stock #7850, 6–8 weeks, 20–25 g) were used. Seven-week-old nude mice were injected with human melanoma cells (1 × 10^6^/flank/animal). When tumours were palpable (50–100 mm^3^), mice were randomly divided into four cohorts and fed vehicle, BRAFi/MEKi (PLX4720 200 ppm + PLX2695 7 ppm), BETi (PLX51107 90 ppm) or BRAFi/MEKi/BETi-laced chows (Research Diets Inc., New Brunswick, NJ) in a scheduled treatment, as indicated. All cohorts had six animals (three females, three males). For BRAF-mutant PDX TJUMEL41 experiments, highly immunodeficient NOD-SCID IL2Rγ^null^ (NSG) mice (6–8 weeks, 20–25 g) were used. BRAF-mutant human melanoma biopsy (PDX TJUMEL41) was collected and processed as previously described.^13,29^ Briefly, NSG mice were anaesthetised with isoflurane inhalation. The area was scrubbed with ethanol, shaved, and a small incision (~5 mm) was made. Human melanoma tissues (collaboration with Dr. Adam C. Berger, TJU Surgical Oncology) were then implanted subcutaneously, and the incisions were closed with wound clips. Mice were observed until they  awoke from anaesthesia, and the wound clips were removed after 7 days. Animals were monitored for any sign of pain such as anorexia, dehydration, hunched posture, failure to groom, excessive redness, and swelling at the incision site. A dose of carprofen or buprenorphine was administered pre-op and as needed from animal monitoring until staple removal. When tumours were palpable, mice were randomly sorted into four cohorts and treated as indicated. All cohorts had seven animals (two females, five males), except for the BETi group (five females). Tumour volume measurements, animal behaviour, and survival were monitored every 2–3 days once treatments began. Digital calliper measurements were used to calculate tumour volumes using the formula: volume = (length × width^2^)/2. When approximately 20% of body weight loss was observed, DietGel 76 A (Scanbur) was administrated. Animals were sacrificed when tumours reached ~650 mm^3^, for 1205Lu and A375 xenografts, or ~1.5 cm^3^, for PDX models. Animals that progressed in weight loss were also sacrificed and included in overall survival results. We used CO_2_ inhalation as our euthanasia method, performed in accordance with the AVMA Panel on Euthanasia for adult animals. This information can also be found in items 7, 8, and 10 of ARRIVE checklist (Supplementary Section).

### Statistical analysis

Correlation analysis was performed to determine the association between either BRD2 or BRD4, and ERBB3 gene and protein expression in BRAF mutant using the TCGA cutaneous melanoma dataset. The TCGA SKCM replicate-based normalised RPPA data (v 4.0) were collected from The Cancer Proteome Atlas (TCPA, http://tcpaportal.org/tcpa/),^30^ while the RNA-seq V2- normalised gene expression and mutation call data were retrieved from the latest Broad GDAC Firehose data run (stddata__2016_01_28, https://confluence.broadinstitute.org/display/GDAC/Home). Spearman’s correlation analysis was performed using the corr() function in Matlab®. In vitro data were expressed as mean ± standard deviation and statistically analysed using Student’s *t*-test (two-tailed, unpaired, and assumed unequal variance) with *p* < 0.05. Means and standard deviations were calculated using experimental triplicates from three separate assays. In vivo statistical analysis is described in item 9 of ARRIVE checklist (Supplementary Section).

## Results

### BRD/BET inhibitors block adaptive RTK upregulation following BRAFi/MEKi treatment

**Fig. 1:**
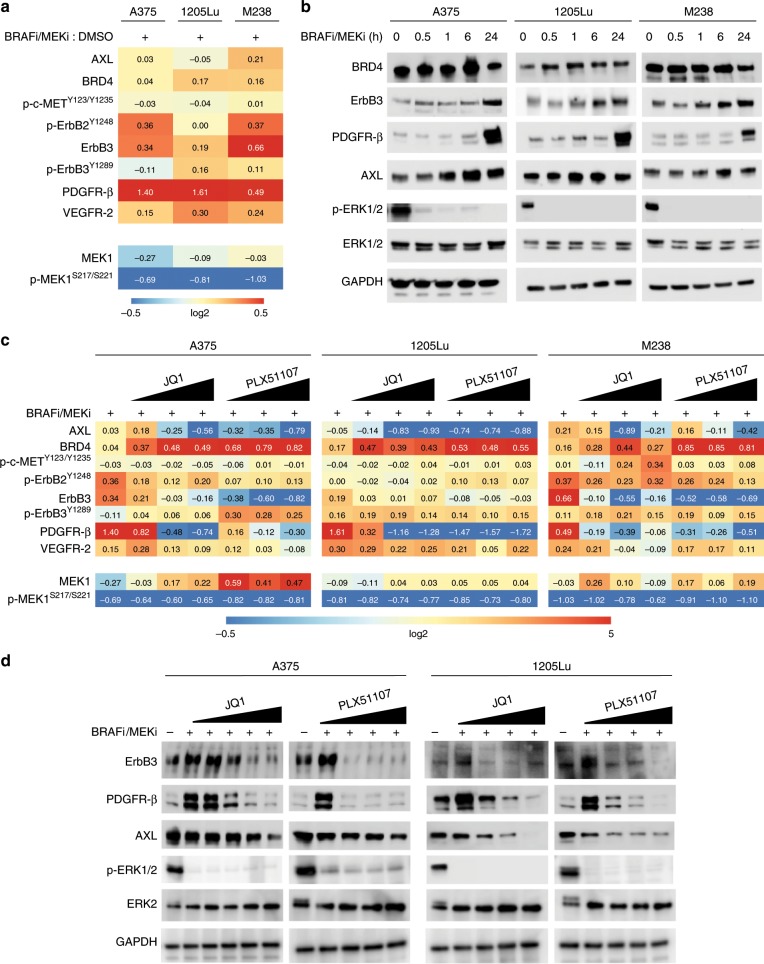
BET inhibition represses adaptive RTK expression following BRAF/MEK inhibitors treatment in BRAF-mutant melanoma. **a** RPPA analysis for the BRAF-mutant human melanoma cell lines, A375, 1205Lu, and M238, after 24 h of treatment with BRAFi/MEKi (dabrafenib, 50 nM + trametinib, 5 nM). RPPA scores for proteins involved in RTK signalling that were statistically significant (*p* < 0.05) with BRAFi/MEKi versus vehicle (DMSO) treatment samples. **b** Western blots for RPPA-identified proteins in **a** after treatment with BRAFi/MEKi over a 24-h time course. **c** RPPA analysis for the cell lines, A375, 1205Lu, and M238, after 24 h of treatment with BRAFi/MEKi (dabrafenib, 50 nM + trametinib, 5 nM) ± BETi in a dose-dependent manner (either JQ1: 0.01, 0.1, 1, and 5 μM or PLX51107: 1, 2, and 4 μM). Shown are antibodies for proteins involved in RTK signalling found to be statistically significant (*p* < 0.05) in at least one comparison between BRAFi/MEKi ± BETi-treated groups. **d** Representative Western blots of three (*n* = 3) independent experiments for RTK proteins after 24 h of combination treatment with BRAFi/MEKi ± BETi in a dose-dependent manner for the doses described above.

RTKs are implicated in adaptive responses to BRAFi/MEKi treatment.^7,14,31,32^ By RPPA analysis, PDGFR-β and ErbB3 were upregulated in BRAF-mutant human melanoma cell lines, A375, 1205Lu, and M238 treated with BRAFi/MEKi for 24 h (Fig. 1a). In addition, phospho-ErbB2 and VEGFR-2 were upregulated in two of the three cell lines. By contrast, AXL expression was only modestly increased or unaffected following BRAFi/MEKi treatment. RPPA findings for PDGFR-β, ErbB3, and AXL were confirmed by Western blot for all cell lines (Fig. 1b). To determine the role of BRD/BET proteins, we tested the effects of a new BETi following BRAFi/MEKi short treatment. PLX51107 is a BETi with a short half-life that is structurally distinct from other members of this class of inhibitors and has entered a Phase 1b/2 trial for solid malignancies (Clinicaltrials.gov: NCT02683395). For comparison, we used JQ1, a first-generation BETi.^33^ By RPPA, both JQ1 and PLX51107 significantly reduced the expression of multiple RTKs including those upregulated by BRAFi/MEKi (Fig. 1c). Western blot confirmed that JQ1 and PLX51107 reduced the upregulation of PDGFR-β and ErbB3 by BRAFi/MEKi, as well as basal AXL expression in a dose–dependent manner (Fig. 1d). BRAFi/MEKi/BETi blocked adaptive ERBB3 and PDGFRB gene expression, when compared with BRAFi/MEKi treatment (Supplementary Fig. 1). These data indicate that BETi block upregulated compensatory RTK expression associated with adaptive/intrinsic resistance to BRAFi/MEKi treatment. The structures for inhibitors used in this paper can be found in Supplementary Fig. 2.

### BET inhibitors suppress ErbB3 activation following BRAFi/MEKi treatment

**Fig. 2:**
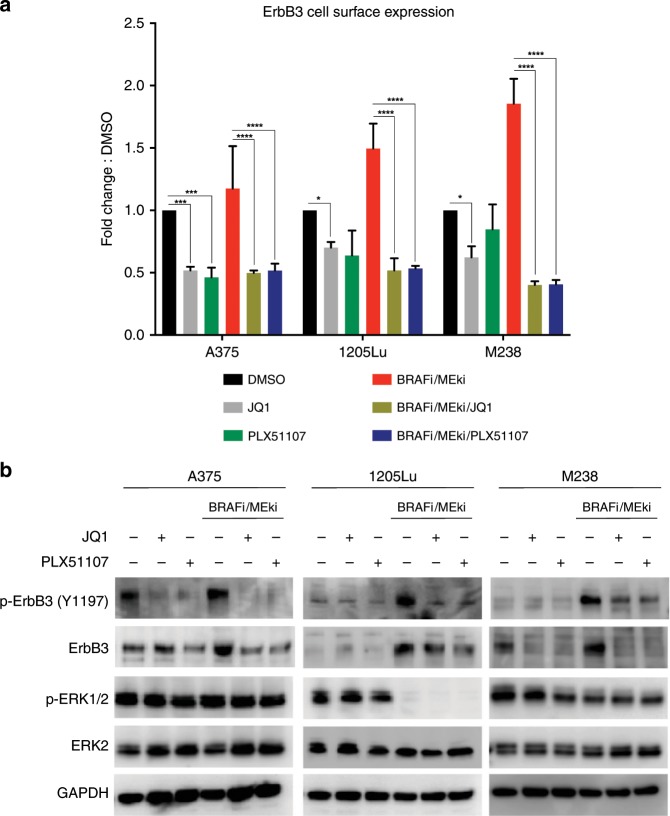
BET inhibition suppresses adaptive ErbB3 activation following BRAF/MEK inhibitors treatment in BRAF-mutant melanoma. **a** ErbB3 cell surface expression after 24 h of combination treatment with BRAFi/MEKi (dabrafenib, 50 nM + trametinib, 5 nM) ± BETi (either JQ1, 1 μM or PLX51107, 2 μM). Tukey’s test (*p < 0.05, ***p < 0.001, ****p < 0.0001). **b** Representative Western blots of three (*n* = 3) independent experiments for ErbB3 activation after combination treatment using BRAFi/MEKi ± BETi (JQ1, 1 μM or PLX51107, 2 μM) for 24 h and neuregulin-1 (NRG1, 10 ng/mL) for 15 min.

Since BETi blocked ErbB3 protein expression, we next tested the effect of BETi on ErbB3 cell surface levels and activation. Both JQ1 and PLX51107 reduced ErbB3 cell surface expression, as detected by flow cytometry, in BRAFi/MEKi-treated A375, 1205Lu, and M238 cells (Fig. 2a). The addition of BETi following BRAFi/MEKi also suppressed the active form of ErbB3 (phospho-ErbB3 Y1197), when cells were stimulated with neuregulin-1 (NRG1) (Fig. 2b). Re-activation of ERK1/2 was observed in two out of three of these lines after 15 min, but this effect was very transient and dissipated after 60 min. These data indicate that BETi reduce the activation of a RTK that is upregulated following BRAFi/MEKi treatment in melanoma.

### BRD2/4 are required for BRAFi/MEKi-associated ErbB3 upregulation in BRAF-mutant melanoma

**Fig. 3:**
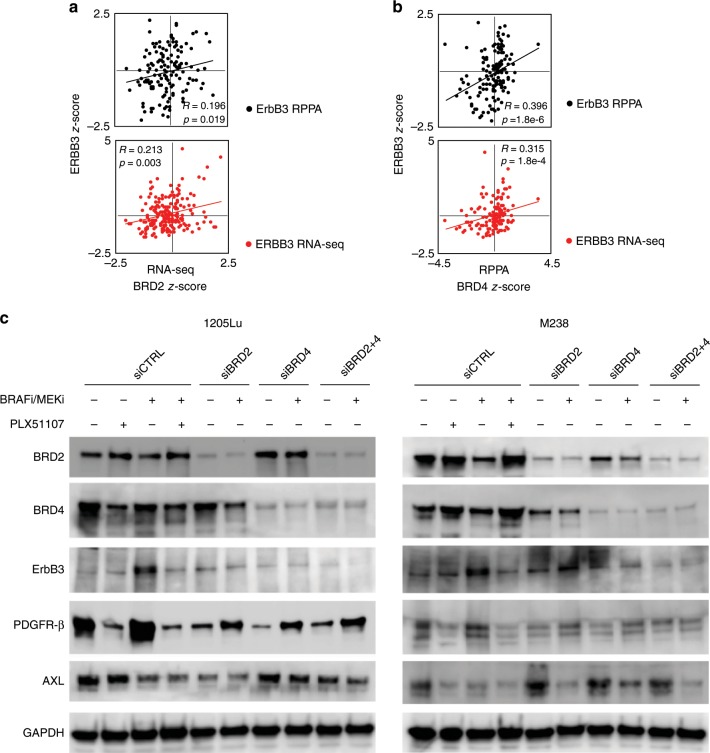
BRD/BET proteins are required for adaptive ErbB3 upregulation following BRAF/MEK inhibitors treatment in BRAF-mutant melanoma. **a** Scatter plots with trend lines of BRD2 mRNA versus ERBB3 mRNA z-score data from BRAF-mutant TCGA cutaneous melanoma samples (*n* = 142). **b** Scatter plots with trend lines of BRD4 mRNA versus ERBB3 representative Western blots of three (*n* = 3) independent experiments for ErbB3, PDGFR-β, and AXL after BRD2 and BRD4 knockdown, and **c** 24 h of treatment using BRAFi/MEKi (dabrafenib, 50 nM + trametinib, 5 nM) for the BRAF-mutant human melanoma cell lines, 1205Lu and M238. The siRNA scramble control (siCTRL), BETi (PLX51107, 2 μM) single agent, and BRAFi/MEKi/BETi treatments for 24 h were used as controls.

As BETi efficiently targeted ErbB3 expression in response to BRAFi/MEKi, we examined the correlation between BRD/BET expression and ErbB3 levels/phosphorylation in BRAF V600E/K-mutant cutaneous melanoma.^34^ In TCGA datasets, BRD2 and BRD4 levels were significantly correlated with ERBB3 gene and protein expression levels in BRAF-mutant melanoma patients (Fig. 3a, b, and Supplementary Fig. 3A). ERBB3 showed statistically significant positive correlations with BRD2 and BRD4 at both RNA and protein levels. No correlation was observed either between BRD2 or BRD4  and PDGFRB transcript and protein levels (Supplementary Fig. 3B, C). The count of samples harbouring different BRAF mutations, as well as the correlation results for BRAFp.V600E/M/G patient samples are provided in Supplementary Fig. 3D, E. Although the link of expression/depletion of BRD2/3/4 was previously described,^35^ we did not find any correlation between BRD3 and PDGFRB or ERBB3 in the TCGA cutaneous melanoma dataset (Supplementary Fig. 4).

We examined the requirement for individual BRD/BET proteins in regulating the adaptive response to BRAF/MEK inhibition. We targeted BRD2 and BRD4 family members given their abundant expression in melanoma.^17^ Individual siRNAs to either BRD2 or BRD4 selectively reduced expression of their target protein (Fig. 3c). ErbB3 expression was dependent on both BRD4 and BRD2 expression in 1205Lu and M238 cells, although the effects of BRD2 knockdown were partial in M238 cells. Adaptive PDGFR-β induction following BRAFi/MEKi treatment was also dependent, at least in part, on both BRD2 and BRD4. The effects on AXL were heterogeneous. AXL expression was reduced by BRAFi/MEKi treatment in both cell lines by PLX51107 treatment and BRD2 knockdown in 1205Lu cells. Since BRD4 encodes two different isoforms, short (BRD4 S) and long (BRD4 L),^36^ we used another antibody to evaluate the isoform expression levels of BRD4 following BRD2/4 targeting. We found that both BRD4 S and BRD4 L isoforms were downregulated in 1205Lu cells after BET knockdown. BRD4 S was not expressed in M238 cells (Supplementary Fig. 5). These data suggest that both BRD2 and BRD4 are required to regulate ErbB3 and PDGFR-β induction following BRAFi/MEKi in BRAF-mutant melanoma.

### BET inhibition enhances the effect of BRAFi/MEKi on cell proliferation in BRAF V600E melanoma cell lines

**Fig. 4:**
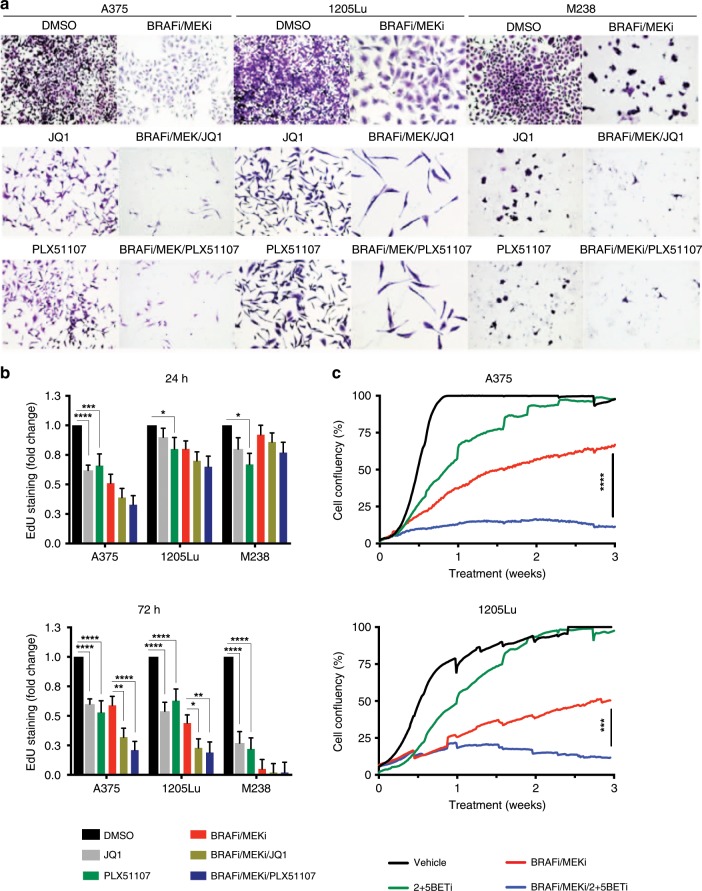
BET inhibitors enhance the effects of BRAF/MEK inhibitors in BRAF-mutant melanoma. **a** Colony assay for BRAF-mutant human melanoma cell lines, A375, 1205Lu, and M238 after 2 weeks of combination treatment using BRAFi/MEKi (dabrafenib, 50 nM + trametinib, 5 nM) ± BETi (either JQ1, 1 μM or PLX51107, 2 μM). Magnification: ×20. **b** S-phase cell cycle arrest detection by EdU staining for A375, 1205Lu and M238 cells after treatment with BRAFi/MEKi ± BETi over a time course (24–72 h). Tukey’s test (*p *<* 0.05, ***p < 0.001, ****p < 0.0001). **c** Cell proliferation curves by IncuCyte® assay over 3 weeks of combination treatment using BRAFi/MEKi ± BETi (PLX51107, 2 μM) for the cell lines A375 and 1205Lu. Tukey’s test (***p < 0.001, ****p < 0.0001).

Next, we tested the effects of BETi on cell proliferation in vitro. In human BRAF V600E melanoma cell lines, JQ1 and PLX51107 treatment alone partially reduced colony growth (Fig. 4a). Importantly, both JQ1 and PLX51107 improved the growth inhibitory effects of BRAFi/MEKi treatment in A375, 1205Lu, and M238 cells. Analysis of 5-ethynyl-2 deoxyuridine (EdU) incorporation showed that BET inhibition alone reduced S-phase entry (Fig. 4b). Furthermore, the triple combination of BRAFi/MEKi/BETi further enhanced cell cycle arrest compared with BRAFi/MEKi, with effects being evident after 72 h of treatment in A375 and 1205Lu cells. M238 cells were highly sensitive to BRAFi/MEKi treatment after 72 h and did not show additional responses to BETi. Similar effects were observed with both JQ1 and PLX51107. We also evaluated targeting BET proteins in combination with BRAFi/MEKi in long-term in vitro assays. After 3 weeks of continuous treatment using BRAFi/MEKi/BETi, A375 and 1205Lu cells showed reduced growth compared with either BRAFi/MEKi or BETi alone (Fig. 4c). Those data suggest that BRAFi/MEKi/BETi treatment prolonged the effects of tumour inhibition, in addition to blocking adaptive upregulation of RTKs by BRAFi/MEKi, suggesting that BETi could avoid targeted therapy-tolerant cell growth.

**Fig. 5:**
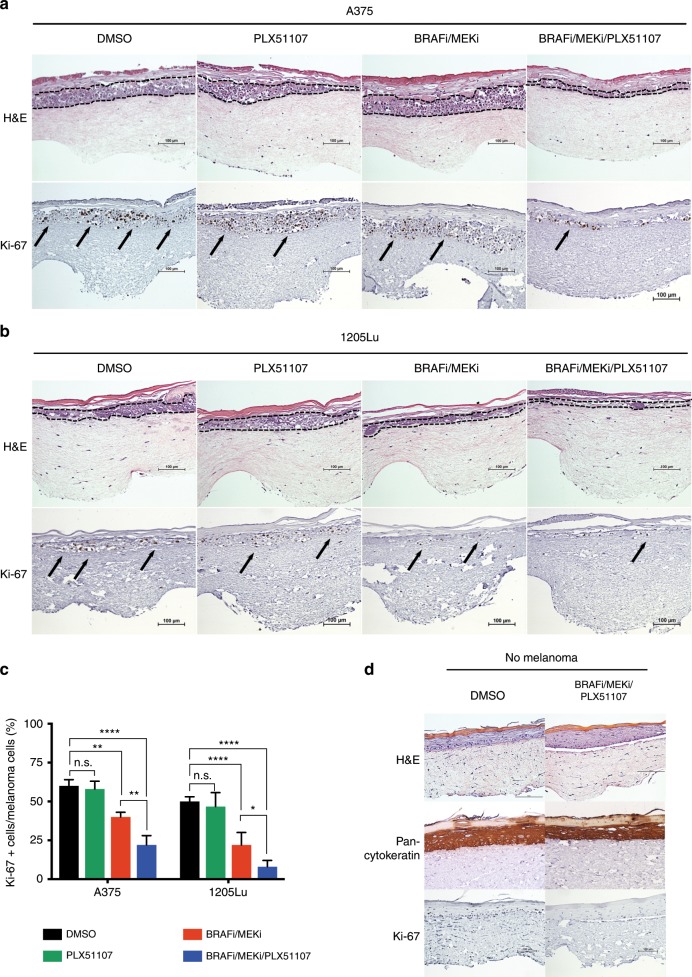
BET inhibitors enhance the effects of BRAF/MEK inhibitors in BRAF-mutant melanoma in a human reconstruct skin tumour microenvironment. Human skin reconstructs containing the BRAF-mutant human melanoma cell lines, either **a** A375 or **b** 1205Lu, after 2 weeks of skin differentiation/melanoma invasion and 48 h of combination treatment using BRAFi/MEKi (dabrafenib, 50 nM + trametinib, 5 nM) ± BETi (PLX51107, 2 μM). Staining: haematoxylin and eosin (H&E), and Ki-67. The dashed lines delimited melanoma invasion area and the arrows indicate melanoma cells. Scale bars: 100 µm. Magnification: ×200. **c** Quantification of Ki-67 staining in melanoma cells for the conditions above. Tukey’s test (*p < 0.05, **p < 0.01, ***p < 0.001, ****p < 0.0001, and n.s.: non-significant) **d** Skin reconstruct control with no melanoma cells. Scale bars: 100 µm. Magnification: ×200.

Since the stromal microenvironment is known to protect melanoma cells from drug therapy,^13,27,37^ we investigated BETi effects on melanoma cell growth in a 3D human skin mimetic model. This model incorporates human keratinocytes that form an epidermal-like layer on a fibroblast-containing dermal-like compartment. BRAFi/MEKi/PLX51107 treatment reduced staining for Ki-67 compared with either PLX51107 alone or BRAFi/MEKi in A375 melanoma cells (Fig. 5a). Similar effects were observed when 1205Lu cells were utilised in this model (Fig. 5b). Quantitation of Ki-67 staining showed a significant reduction with the triple combination compared with BRAFi/MEKi treatment (Fig. 5c). The triple-inhibitor treatment did not dramatically alter normal keratinocyte cell proliferation and differentiation in human skin reconstructs (Fig. 5d). These data indicate that BETi may enhance the effects of BRAFi/MEKi on reducing melanoma cell growth.

### Intermittent BETi schedule enhances durable responses in combination with BRAFi/MEKi

To determine whether BETi treatment may contribute to improved therapeutic outcomes in BRAF-mutant melanoma in vivo, we compared the triple combination of BRAFi/MEKi/BETi with BRAF/MEK targeting in xenograft models (Supplementary Fig. 6A). Initial experiments testing continuous BRAFi/MEKi/BETi treatment in tumour-bearing mice had to be interrupted early in treatment (before day 24) due to symptoms of drug toxicity, including lethargic behaviour and ≥20% of body weight loss in treated animals (Supplementary Fig. 6B–D).

**Fig. 6:**
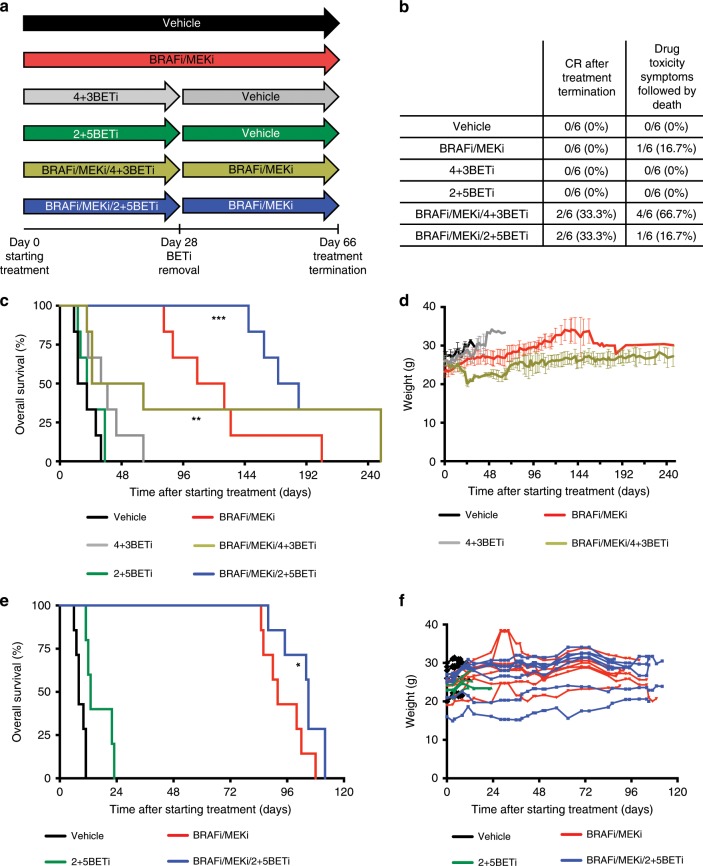
BRAF/MEK/BET inhibitors scheduling delays tumour relapse and prolongs survival in vivo. **a** Treatment schedule schema for 4 days on/3 days off BETi chow (4 + 3BETi) + vehicle or with continuous BRAFi/MEKi chow(BRAFi/MEKi/4 + 3BETi), and 2 days on/5 days off BETi chow (2 + 5BETi) + vehicle or with continuous BRAFi/MEKi chow (BRAFi/MEKi/2 + 5BETi) in a 7-day cycle for 4 cycles, compared with vehicle or continuous BRAFi/MEKi-alone. After day 28, BETi was removed and animals were maintained either in vehicle or BRAFi/MEKi chow. On day 66, the remaining mice bearing 1205Lu xenografts were switched to control chow to evaluate residual disease. **b** Percentage of 1205Lu xenograft-bearing mice that presented with a complete response (CR) after cessation of therapy and symptoms of drug toxicity. **c** 1205Lu xenograft mice overall survival (time-to-event end point: death or tumour size reaches ~650 mm^3^). Kaplan–Meier analysis (**p < 0.01, ***p < 0.001). **d** 1205Lu xenograft mice weight loss for BRAFi/MEKi/4 + 3BETi scheduling group during drug treatment. **e** BRAF-mutant PDX TJUMEL41-bearing mice overall survival (time-to-event end point: death or tumour size reaches ~1.5 cm^3^). Kaplan–Meier analysis (*p < 0.05). **f** PDX TJUMEL41-bearing mice weight measurements during drug treatments.

To manage toxicity while retaining efficacy, we tested BETi intermittent dosing schedules combined with continuous BRAF/MEK targeting. Intermittent schedules in vivo were based on in vitro studies suggesting that either 4 days on/3 days off BETi (4 + 3BETi) or 2 days on/5 days off BETi (2 + 5BETi) would prolong BRAF/MEK targeting effects (Supplementary Fig. 6E). We utilised intermittent scheduling of 4 days on/3 days off BETi combined with continuous BRAFi/MEKi (BRAFi/MEKi/4 + 3BETi) in 7-day cycles for 4 cycles followed by continuous BRAFi/MEKi until 66 days of treatment (Fig. 6a). After day 66, treatments were stopped, and residual disease investigated. We observed a higher CR rate in animals treated with BRAFi/MEKi/4 + 3BETi lead-in versus BRAFi/MEKi alone (33% vs. 0%, Fig. 6b). Prolonged CRs after treatment cessation were observed only for BRAFi/MEKi/4 + 3BETi treatment (Figs. 6b, c and Supplementary Fig. 7A). Although fewer toxicities were observed with BRAFi/MEKi/4 + 3BETi compared with continuous triple-inhibitor treatment, 67% of the animals still presented symptoms of drug toxicity and severe weight loss (Fig. 6d and Supplementary Fig. 7B). Use of an intermittent scheduling of BRAFi/MEKi for 7 days followed by BETi alone for 7 days in 2 cycles was effective in vitro (Supplementary Fig. 6E); however, this schedule for 24 days followed by continuous BRAFi/MEKi until day 66 lacked efficacy in vivo (Supplementary Fig. 7C–F).

Due to observed effects in vitro (Supplementary Fig. 6E), we tested a reduced 2 days on/5 days off schedule of BETi combined with continuous BRAFi/MEKi (BRAFi/MEKi/2 + 5BETi) in 7-day cycles for 4 cycles. At this time, BETi was removed and animals were maintained in either vehicle or BRAFi/MEKi chow. Again, we investigated regrowth of disease after cessation of treatment. BRAFi/MEKi/2 + 5BETi led to a 33% CR without affecting weight loss or the animals presenting any symptoms of drug toxicity (Fig. 6b and Supplementary Fig. 7B). Importantly, the addition of BETi following BRAFi/MEKi delayed tumour recurrence after cessation of BRAFi/MEKi treatment and improved animal survival outcome (Fig. 6c, d). Similar results were found in A375 xenografts using the BRAFi/MEKi/2 + 5BETi schedule, which led to a 33% CR without affecting weight loss or presenting any symptoms of drug toxicity (Supplementary Fig. 8A, B). Furthermore, the BRAFi/MEKi/2 + 5BETi schedule delayed tumour growth in a BRAF-mutant melanoma patient-derived xenograft (PDX) model (Supplementary Fig. 8C, D). These effects were associated with a significantly prolonged animal survival (Fig. 6e) without affecting weight loss (Fig. 6f). Together, these data indicate that use of an intermittent BETi schedule may be tolerable and enhances the effects of BRAFi/MEKi in BRAF-mutant melanoma.

## Discussion

The duration of responses to targeted inhibitors in BRAF-mutant melanoma is limited by rapid adaptations that select for drug-tolerant cell subpopulations. Here, we utilised a new BETi, PLX51107, in combination with BRAFi/MEKi in BRAF-mutant melanoma models to broadly suppress adaptive upregulation of RTKs in response to targeted therapy. The choice of those melanoma cell lines was based on previous studies where adaptive upregulation of ErbB3 and PDGFR-β was observed following BRAF targeting, and those RTKs were associated with resistance to targeted therapy.^16,20^ Importantly, we show that an intermittent dosing schedule of PLX51107 was tolerable and prolonged the growth inhibition achieved by continuous BRAFi/MEKi treatment.

Multiple RTKs are upregulated in BRAF-mutant melanoma cells following BRAFi/MEKi^38^ and may be activated by growth factors derived from the stromal microenvironment.^1,13^ We show that the next-generation BETi, PLX51107, inhibited BRAFi/MEKi-induced upregulation of two RTKs, ErbB3 and PDGFR-β. These findings are conceptually similar to evidence that the short-term inhibition of BET proteins using JQ1, I-BET151, or I-BET762 reduced lapatinib resistance that was associated with adaptive kinome expression in triple-negative breast cancer models.^23,39^ In these contexts, BETi reduced adaptive RTK expression followed by RTK-associated focal adhesion kinase, Src family kinase phosphorylation and AKT re-activation.^18,23,24,40^ BETi preferentially inhibit targeted therapy-induced gene expression, since JQ1 downregulated ~8% of all expressed genes when used alone but suppressed 27% of lapatinib-induced genes in combination treatment.^23,41,42^ Together, these data suggest that BETi will be useful to suppress adaptive RTK upregulation and enhance tumour inhibitory effects by BRAFi/MEKi therapy.

BETi also regulate the expression of targets other than RTKs. BETi were first identified as a c-Myc and histone modification-targeting therapy.^43^ Our data show that BETi enhanced the cell cycle arrest induced by BRAFi/MEKi after 48 h of treatment, consistent with studies that showed that BRD/BET proteins regulate expression of p21(WAF1/Cip1) and c-Myc.^17,44^ Furthermore, the concomitant targeting of PI3K with JQ1 resulted in potent tumour growth arrest and apoptosis in ovarian cancer cells, compared with either agent alone.^45^ Although BRAF/MEK targeting reduced ERK1/2 pathway activation and cell survival, therapy-tolerant cell subpopulations may persist and seed disease relapse.^46^ Residual cells have presented a mesenchymal-like cell shape, prominent actin stress fibres, high invasiveness, low proliferation, and neural crest cell markers;^32,46^ targeting these drug-tolerant cells using BETi may avoid adaptive response and delay melanoma relapse.^47,48^ Blockage of adaptive BRD/BET-induced upregulation of ErbB3/PDGFR-β following BRAFi/MEKi therapy may contribute to the effects of BETi on delayed tumour recurrence, since those RTKs were closely associated with targeted therapy resistance and poor survival in BRAF-mutant melanoma patients.^11,49^ However, we do not rule out a role for other BETi targets. In addition to BRAFi/MEKi-associated RTK downregulation, BETi also altered the cell cycle, contributing to tumour growth inhibition.

We evaluated the therapeutic effects of BETi/BRAFi/MEKi in vivo using human melanoma cell xenografts and PDX models. Continuous dosing of BRAFi/MEKi/BETi was toxic for tumour-bearing mice, and both BRAFi/MEKi and BETi cause toxicities in patients.^50^ Using a continuous triple-schedule combination in mice produced enhanced toxicities, limiting the efficacy of therapy in vivo. Thus, we tested and optimised an intermittent dosing schedule of BETi to enhance BRAFi/MEKi treatment in vivo while maintaining tolerability. While focusing on maximising the short-term effect of BRAFi/MEKi, we also assessed the ability to delay/prevent tumour relapse after cessation of BRAFi/MEKi treatment by monitoring residual disease. Importantly, we observed that BETi following BRAFi/MEKi treatment delayed tumour relapse. We tested several intermittent schedules of BETi to prolong BRAFi/MEKi effects. We observed that an intermittent schedule of 2 days on followed by 5 days off BETi in the presence of BRAFi/MEKi presented less toxicity compared with 4 days on followed by 3 days off BETi schedule in the presence of BRAFi/MEKi, and was more effective than the alternative schedule of 7 days on BRAFi/MEKi alone followed by 7 days on BETi alone. This latter schedule may have been less effective due to the rapid BRAFi/MEKi-associated upregulation of RTKs,^51^ which may require BETi to be administered at the same time of BRAFi/MEKi for efficacy. Although our data showed that BETi enhanced the efficacy of targeted therapies in two different xenografts and one PDX model, a limitation is the small number of animals used in our study. Altogether, our data suggest that co-targeting of BRD/BET proteins in concert with BRAF/MEK inhibitors is likely to promote more durable responses of targeted therapy for BRAF-mutant melanoma in vivo.

## Supplementary information

Supplementary Figures

## Data Availability

The completed checklist that contains the reporting of animal research used in this study is found as ARRIVE guidelines checklist at Supplementary Section. All data generated or analysed during this study are included in this published article and its supplementary information files.

## References

[1] Straussman R, Morikawa T, Shee K, Barzily-Rokni M, Qian ZR, Du J (2012). Tumour micro-environment elicits innate resistance to RAF inhibitors through HGF secretion. Nature.

[2] Robert C, Ribas A, Hamid O, Daud A, Wolchok JD, Joshua AM (2018). Durable complete response after biscontinuation of pembrolizumab in patients with metastatic melanoma. J. Clin. Oncol..

[3] Dankort D, Curley DP, Cartlidge RA, Nelson B, Karnezis AN, Damsky WE (2009). Braf(V600E) cooperates with Pten loss to induce metastatic melanoma. Nat. Genet..

[4] Hsieh A. H.-C., Faithfull S., Brown M. P. Risk of cumulative toxicity after complete melanoma response with pembrolizumab. *BMJ Case Rep*. pii: bcr2016218308. 10.1136/bcr-2016-218308 (2017). 10.1136/bcr-2016-218308 PMC5294005 28148549

[5] Sosman Ja, Kim KB, Schuchter L, Gonzalez R, Pavlick AC, Weber JS (2012). Survival in BRAF V600–mutant advanced melanoma treated with vemurafenib. N. Engl. J. Med..

[6] Hodi FS, Chiarion-Sileni V, Gonzalez R, Grob J-JJ, Rutkowski P, Cowey CL (2018). Nivolumab plus ipilimumab or nivolumab alone versus ipilimumab alone in advanced melanoma (CheckMate 067): 4-year outcomes of a multicentre, randomised, phase 3 trial. Lancet Oncol..

[7] Nazarian R, Shi H, Wang Q, Kong X, Koya RC, Lee H (2010). Melanomas acquire resistance to B-RAF(V600E) inhibition by RTK or N-RAS upregulation. Nature.

[8] Flaherty KT, Robert C, Hersey P, Nathan P, Garbe C, Milhem M (2012). Improved survival with MEK inhibition in BRAF-mutated melanoma. N. Engl. J. Med..

[9] Carlino MS, Vanella V, Girgis C, Giannarelli D, Guminski A, Festino L (2016). Cessation of targeted therapy after a complete response in BRAF-mutant advanced melanoma: a case series. Br. J. Cancer.

[10] Das Thakur M, Salangsang F, Landman AS, Sellers WR, Pryer NK, Levesque MP (2013). Modelling vemurafenib resistance in melanoma reveals a strategy to forestall drug resistance. Nature.

[11] Abel EVE, Basile KKJ, Kugel CH, Witkiewicz AK, Le K, Amaravadi RK (2013). Melanoma adapts to RAF/MEK inhibitors through FOXD3-mediated upregulation of ERBB3. J. Clin. Invest..

[12] Basile KJ, Abel EV, Dadpey N, Hartsough EJ, Fortina P, Aplin AE (2013). In vivo MAPK reporting reveals the heterogeneity in tumoral selection of resistance to RAF inhibitors. Cancer Res..

[13] Capparelli C, Rosenbaum S, Berger AC, Aplin AE (2015). Fibroblast-derived neuregulin 1 promotes Compensatory ErbB3 receptor signaling in mutant BRAF melanoma. J. Biol. Chem..

[14] Li FZ, Dhillon AS, Anderson RL, McArthur G, Ferrao PT (2015). Phenotype switching in melanoma: implications for progression and therapy. Front Oncol..

[15] Shimizu T, Tolcher AW, Papadopoulos KP, Beeram M, Rasco DW, Smith LS (2012). The clinical effect of the dual-targeting strategy involving PI3K/AKT/mTOR and RAS/MEK/ERK pathways in patients with advanced cancer. Clin. Cancer Res..

[16] Kugel CH, Hartsough EJ, Davies MA, Setiady YY, Aplin AE (2014). Function-blocking ERBB3 antibody inhibits the adaptive response to RAF inhibitor. Cancer Res..

[17] Segura MF, Fontanals-Cirera B, Gaziel-Sovran A, Guijarro MV, Hanniford D, Zhang G (2013). BRD4 sustains melanoma proliferation and represents a new target for epigenetic therapy. Cancer Res..

[18] Zawistowski JS, Bevill SM, Goulet DR, Stuhlmiller TJ, Beltran AS, Olivares-Quintero JF (2017). Enhancer remodeling during adaptive bypass to MEK inhibition is attenuated by pharmacologic targeting of the P-TEFb complex. Cancer Discov..

[19] Padmanabhan B, Mathur S, Manjula R, Tripathi S (2016). Bromodomain and extra-terminal (BET) family proteins: New therapeutic targets in major diseases. J. Biosci..

[20] Shi J, Vakoc CR (2014). The mechanisms behind the therapeutic activity of BET bromodomain inhibition. Mol. Cell..

[21] Vázquez R, Riveiro ME, Astorgues-Xerri L, Odore E, Rezai K, Erba E (2017). The bromodomain inhibitor OTX015 (MK-8628) exerts anti-tumor activity in triple-negative breast cancer models as single agent and in combination with everolimus. Oncotarget.

[22] Baratta MG, Schinzel AC, Zwang Y, Bandopadhayay P, Bowman-Colin C, Kutt J (2015). An in-tumor genetic screen reveals that the BET bromodomain protein, BRD4, is a potential therapeutic target in ovarian carcinoma. Proc. Natl Acad. Sci. Usa..

[23] Stuhlmiller TJ, Miller SM, Zawistowski JS, Nakamura K, Beltran AS, Duncan JS (2015). Inhibition of lapatinib-induced kinome reprogramming in ERBB2-positive breast cancer by targeting BET family bromodomains. Cell Rep..

[24] Nakamura Y, Hattori N, Iida N, Yamashita S, Mori A, Kimura K (2017). Targeting of super-enhancers and mutant BRAF can suppress growth of BRAF -mutant colon cancer cells via repression of MAPK signaling pathway. Cancer Lett..

[25] Berthon C, Raffoux E, Thomas X, Vey N, Gomez-Roca C, Yee K (2016). Bromodomain inhibitor OTX015 in patients with acute leukaemia: a dose-escalation, phase 1 study. Lancet Haematol..

[26] Echevarría‐Vargas IM, Reyes‐Uribe PI, Guterres AN, Yin X, Kossenkov AV, Liu Q (2018). Co‐targeting BET and MEK as salvage therapy for MAPK and checkpoint inhibitor‐resistant melanoma. EMBO Mol. Med..

[27] Sandri S, Faião-Flores F, Tiago M, Pennacchi PC, Massaro RR, Alves-Fernandes DK (2016). Vemurafenib resistance increases melanoma invasiveness and modulates the tumor microenvironment by MMP-2 upregulation. Pharm. Res..

[28] Kemper K, Krijgsman O, Kong X, Cornelissen-Steijger P, Shahrabi A, Weeber F (2016). BRAF V600E kinase domain duplication identified in therapy-refractory melanoma patient-derived xenografts. Cell Rep..

[29] Capparelli C, Purwin TJ, Heilman SA, Chervoneva I, McCue PA, Berger AC (2018). ErbB3 targeting enhances the effects of MEK inhibitor in wild-type BRAF/NRAS melanoma. Cancer Res..

[30] Li J, Lu Y, Akbani R, Ju Z, Roebuck PL, Liu W (2013). TCPA: a resource for cancer functional proteomics data. Nat. Methods.

[31] Basile KJ, Abel EV, Aplin AE (2012). Adaptive upregulation of FOXD3 and resistance to PLX4032/4720-induced cell death in mutant B-RAF melanoma cells. Oncogene.

[32] Ahn A, Chatterjee A, Eccles MR (2017). The slow cycling phenotype: a growing problem for treatment resistance in melanoma. Mol. Cancer Ther..

[33] Filippakopoulos P, Qi J, Picaud S, Shen Y, Smith WB, Fedorov O (2010). Selective inhibition of BET bromodomains. Nature.

[34] Ucar D, Lin D (2015). Amplification of the bromodomain-containing protein 4 gene in ovarian high‑grade serous carcinoma is associated with worse prognosis and survival. Mol. Clin. Oncol..

[35] Lambert J-P, Picaud S, Fujisawa T, Hou H, Savitsky P, Uusküla-Reimand L (2019). Interactome rewiring following pharmacological targeting of BET bromodomains. Mol. Cell..

[36] Wu S-Y, Chiang C-M (2007). The double bromodomain-containing chromatin adaptor brd4 and transcriptional regulation. J. Biol. Chem..

[37] Tiago M, De Oliveira EM, Brohem CA, Pennacchi PC, Paes RD, Haga RB (2014). Fibroblasts protect melanoma cells from the cytotoxic effects of doxorubicin. Tissue Eng. Part A..

[38] Shaffer SM, Dunagin MC, Torborg SR, Torre EA, Emert B, Krepler C (2017). Rare cell variability and drug-induced reprogramming as a mode of cancer drug resistance. Nature.

[39] Shu S, Lin CY, He HH, Witwicki RM, Tabassum DP, Roberts JM (2016). Response and resistance to BET bromodomain inhibitors in triple-negative breast cancer. Nature.

[40] Leonard B, Brand TM, O’Keefe RA, Lee ED, Zeng Y, Kemmer JD (2018). BET inhibition overcomes receptor yyrosine kinase–mediated cetuximab resistance in HNSCC. Cancer Res..

[41] Stratikopoulos EE, Dendy M, Szabolcs M, Khaykin AJ, Lefebvre C, Zhou M-M (2015). Kinase and BET inhibitors together clamp inhibition of PI3K signaling and overcome resistance to therapy. Cancer Cell..

[42] Stuhlmiller TJ, Miller SM, Johnson GL (2016). Epigenetic inhibition of adaptive bypass responses to lapatinib by targeting BET bromodomains. Mol. Cell Oncol..

[43] Doroshow DB, Eder JP, LoRusso PM (2017). BET inhibitors: a novel epigenetic approach. Ann. Oncol..

[44] Gallagher SJ, Mijatov B, Gunatilake D, Tiffen JC, Gowrishankar K, Jin L (2014). The epigenetic regulator I-BET151 induces BIM-dependent apoptosis and cell cycle arrest of human melanoma cells. J. Invest Dermatol..

[45] Wyce A, Matteo JJ, Foley SW, Felitsky DJ, Rajapurkar SR, Zhang X-P (2018). MEK inhibitors overcome resistance to BET inhibition across a number of solid and hematologic cancers. Oncogenesis.

[46] Johnson AS, Crandall H, Dahlman K, Kelley MC (2015). Preliminary results from a prospective trial of preoperative combined BRAF and MEK-targeted therapy in advanced BRAF mutation-positive melanoma. J. Am. Coll. Surg..

[47] Fallahi-Sichani M, Becker V, Izar B, Baker GJ, Lin J-RJ, Boswell SA (2017). Adaptive resistance of melanoma cells to RAF inhibition via reversible induction of a slowly dividing de-differentiated state. Mol. Syst. Biol..

[48] Kleczko EK, Heasley LE (2018). Mechanisms of rapid cancer cell reprogramming initiated by targeted receptor tyrosine kinase inhibitors and inherent therapeutic vulnerabilities. Mol. Cancer.

[49] Shi H, Kong X, Ribas A, Lo RS (2011). Combinatorial treatments that overcome PDGFR -driven resistance of melanoma cells to V600EB-RAF inhibition. Cancer Res..

[50] Welsh SJ, Corrie PG (2015). Management of BRAF and MEK inhibitor toxicities in patients with metastatic melanoma. Ther. Adv. Med Oncol..

[51] Rosell R, Karachaliou N, Morales-espinosa D, Costa C, Molina MA, Sansano I (2013). Adaptive resistance to targeted therapies in cancer. Transl. Lung Cancer Res..

