# Supplementary material for: Targeting BRD/BET proteins inhibits adaptive kinome upregulation and enhances the effects of BRAF/MEK inhibitors in melanoma
Source: Br J Cancer. 2020 Jan 14;122(6):789–800. doi: 10.1038/s41416-019-0724-y (PMC7078299; doi:10.1038/s41416-019-0724-y)
Supplement: Supplementary file 1 — Supplementary Figures [file 41416_2019_724_MOESM1_ESM.pdf]

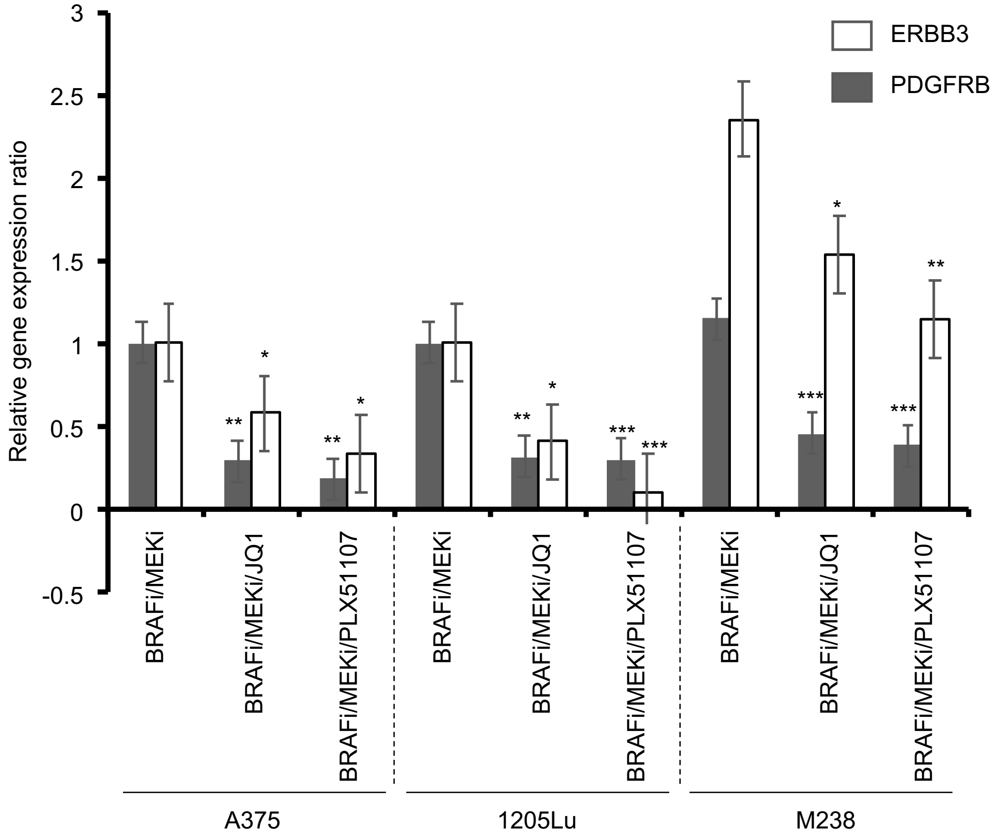

ERBB3
PDGFRB
Relative gene expression ratio
3
2.5
2
1.5
1
0.5
0
-0.5
*
**
***
BRAFi/MEKi
BRAFi/MEKi/JQ1
BRAFi/MEKi/PLX51107
A375
1205Lu
M238

Chemical structures for agents used in this manuscript:

JQ1

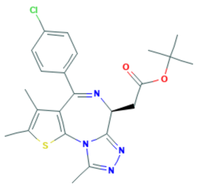

Molecular Formula: $C_{23}H_{25}ClN_4O_2S$

PLX51107

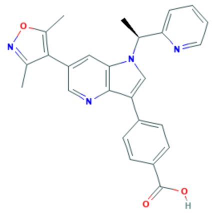

Molecular Formula: $C_{26}H_{22}N_4O_3$

Dabrafenib

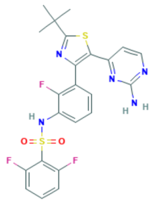

Molecular Formula: $C_{23}H_{20}F_3N_5O_2S_2$

Trametinib

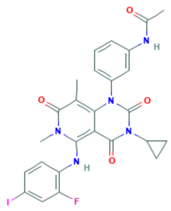

Molecular Formula: $C_{26}H_{23}FIN_5O_4$

PLX4720

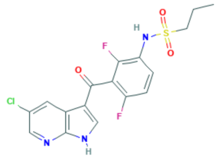

Molecular Formula: $C_{17}H_{14}ClF_2N_3O_3S$

PD0325901

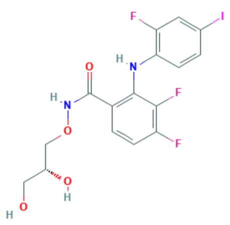

Molecular Formula: $C_{16}H_{14}F_3IN_2O_4$

Source: https://pubchem.ncbi.nlm.nih.gov/

A

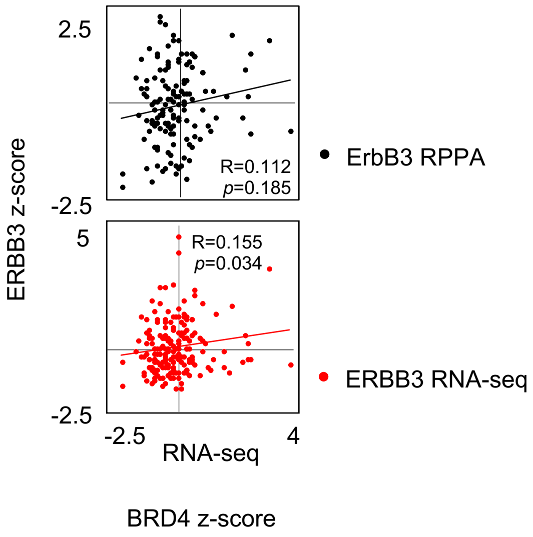

B

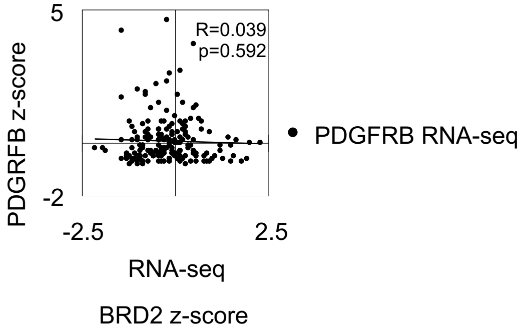

C

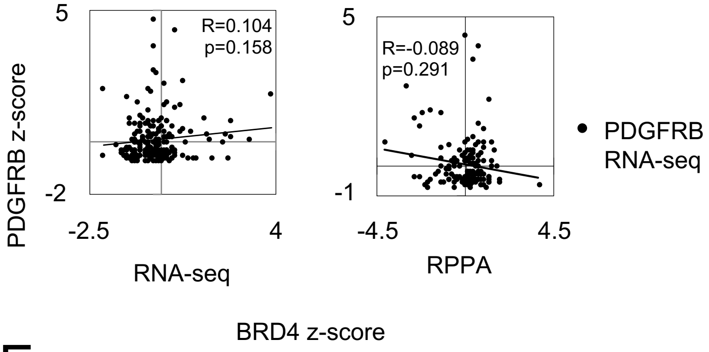

D

Count of patients with each BRAF mutation variant

BRAF mutation	Count of Samples
p.V600E	123
p.G466E/A/R	4
p.V600M/G	3
p.N581H/S/T	3
p.K601E	3
p.S467L	2
p.600_601VK>E	1
p.D594N	1
p.L485F	1
p.P367S	1

E

Spearman’s correlation results for BRAFp.V600E/M/G patients for BRD2 and BRD4 vs ERBB3

BRAF p.V600-mutant only (n=126)		
BRD4 RNA-Seq vs ERBB3		
Gene/Protein	Spearman's r	Spearman p-val
ERBB3_RNASeq	0.160	0.041
ErbB3_rppa	0.155	0.084
BRD4 RPPA vs ERBB3		
Gene/Protein	Spearman's r	Spearman p-val
HER3_rppa	0.3737	1.7E-05
ERBB3_rnaseq	0.3091	0.0004
BRD2 RNA-Seq vs ERBB3		
Gene/Protein	Spearman's r	Spearman p-val
ERBB3_RNASeq	0.232	0.003
ErbB3_rppa	0.156	0.082

Tiago et al., Supplementary Figure 4

A

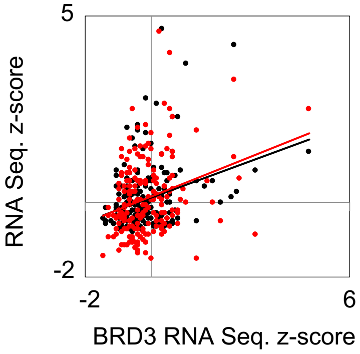

● PDGFRB RNA Seq.
Spearman's r = 0.236
p-value = 0.001

● ERBB3 RNA Seq.
Spearman's r = 0.247
p-value = 6.85 x 10^{-4}

B

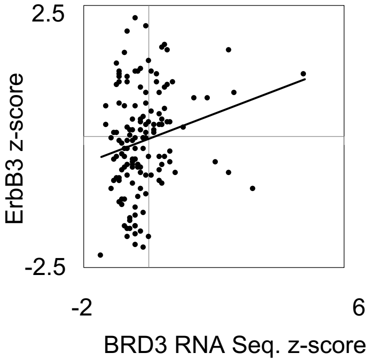

● ErbB3 RPPA
Spearman's r = 0.212
p-value = 0.011

C

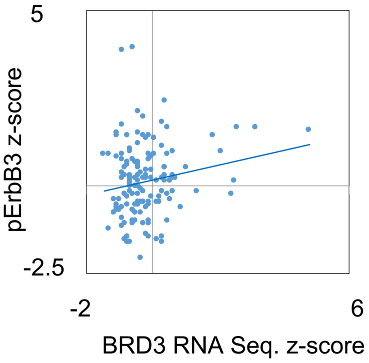

● pErbB3 RPPA
Spearman's r = 0.141
p-value = 0.095

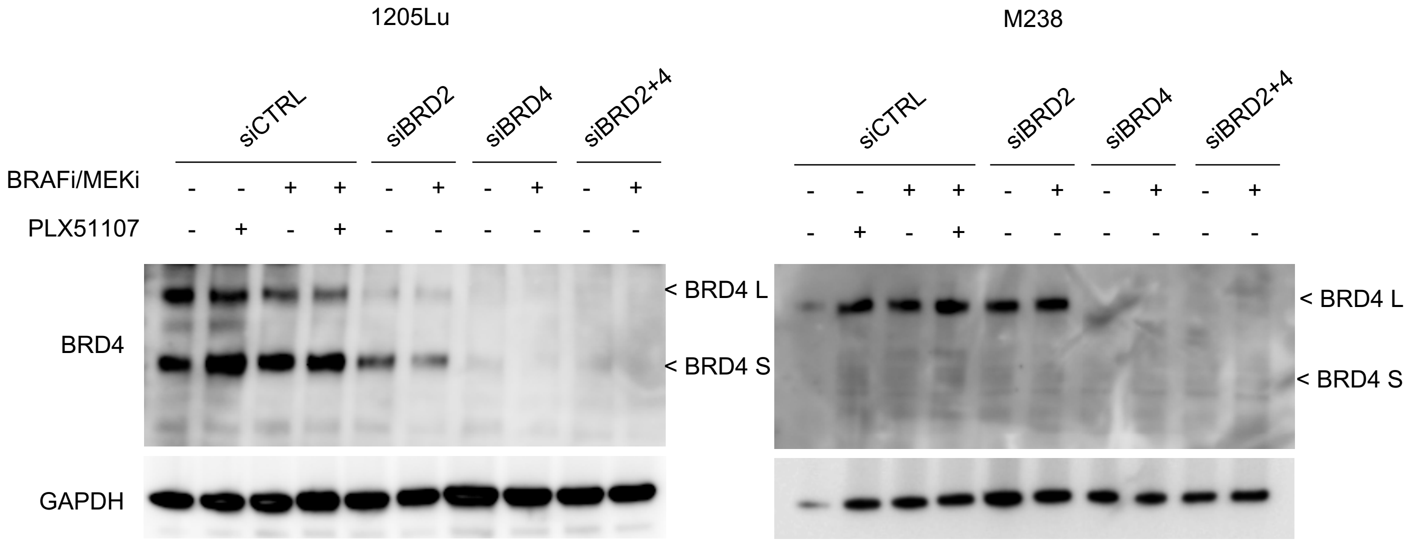
1205Lu
M238
siCTRL
siBRD2
siBRD4
siBRD2+4
BRAFi/MEKi
PLX51107
BRD4
< BRD4 L
< BRD4 S
GAPDH

A

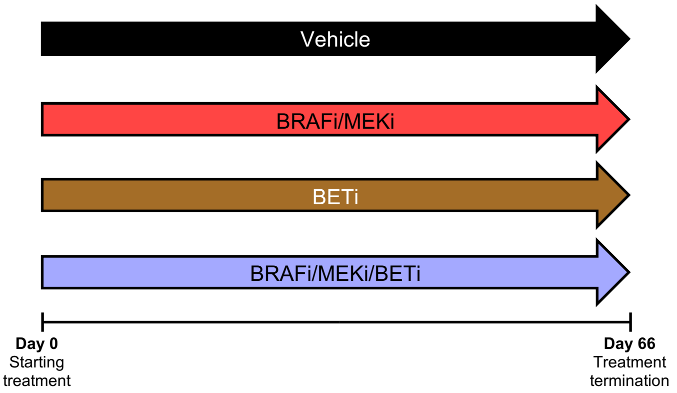

B

	CR after treatment termination	Drug toxicity symptoms followed by death
Vehicle	0/6 (0%)	0/6 (0%)
BRAFi/MEKi	0/6 (0%)	0/6 (0%)
BETi	0/6 (0%)	2/6 (33.4%)
BRAFi/MEKi/BETi	ND	6/6 (100%)

C

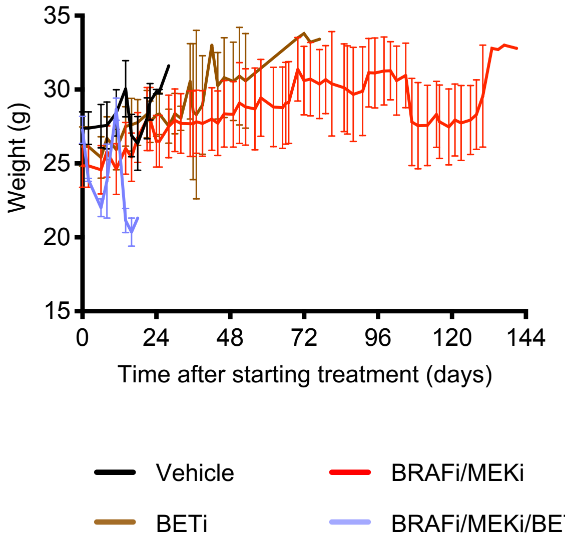

D

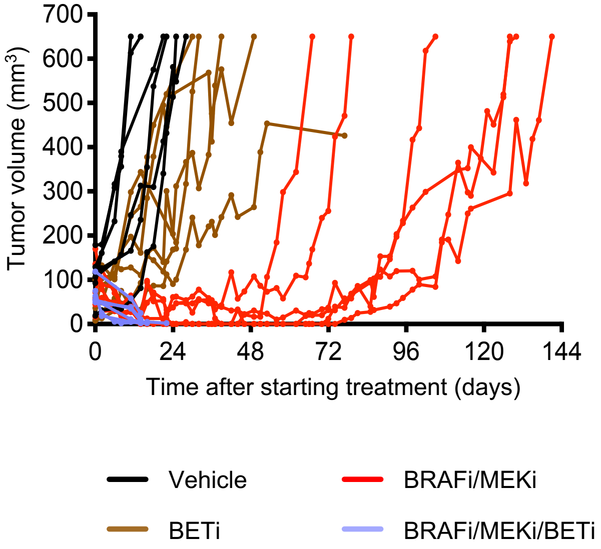

E

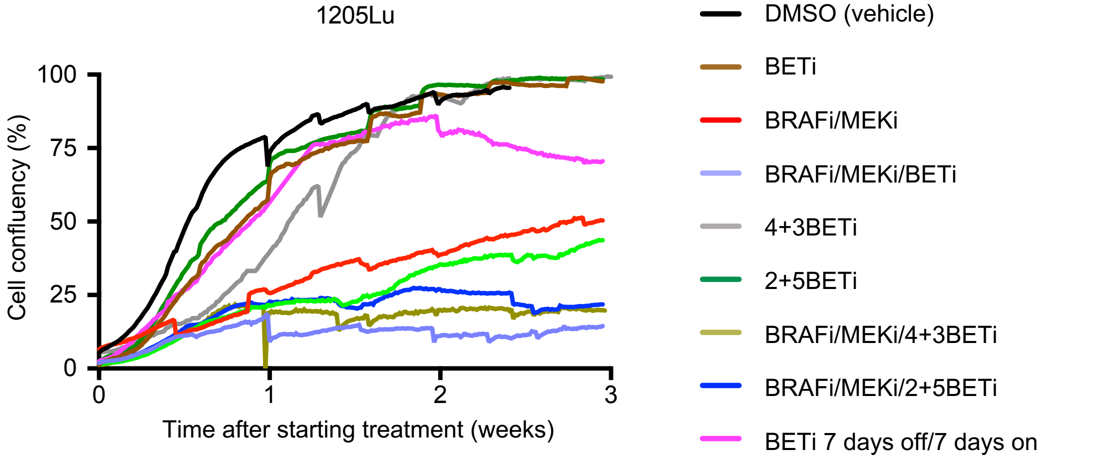

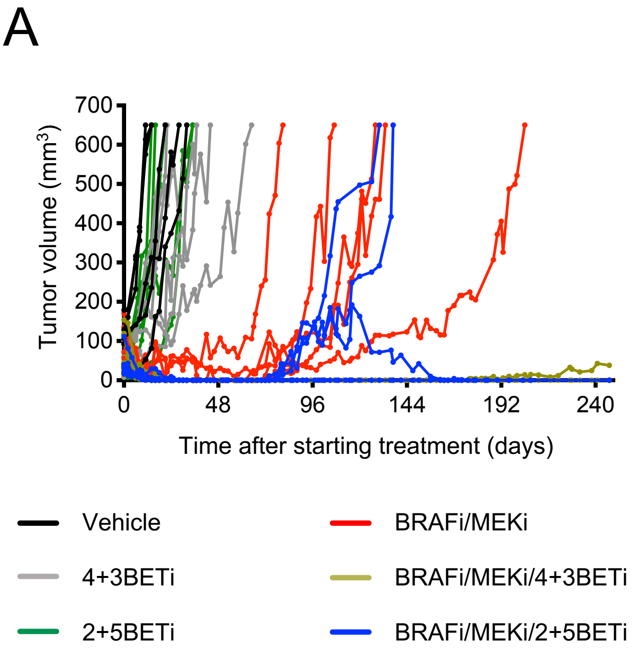

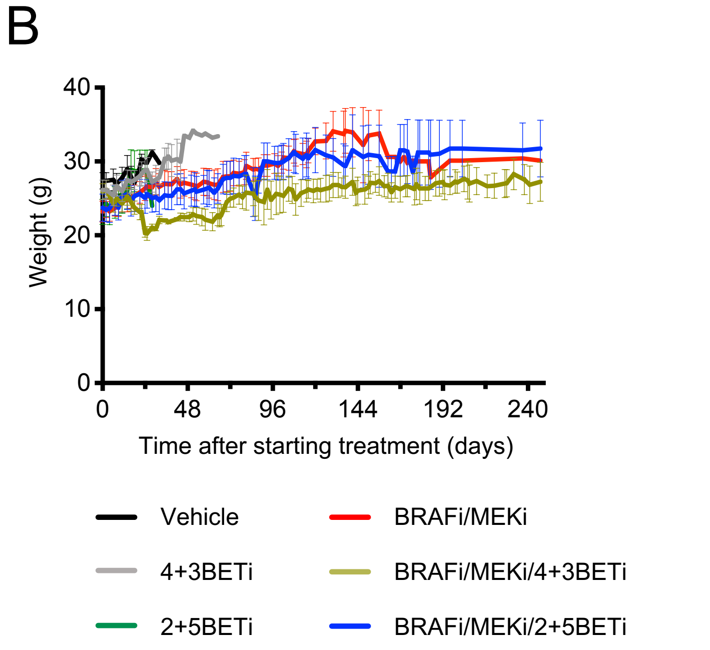

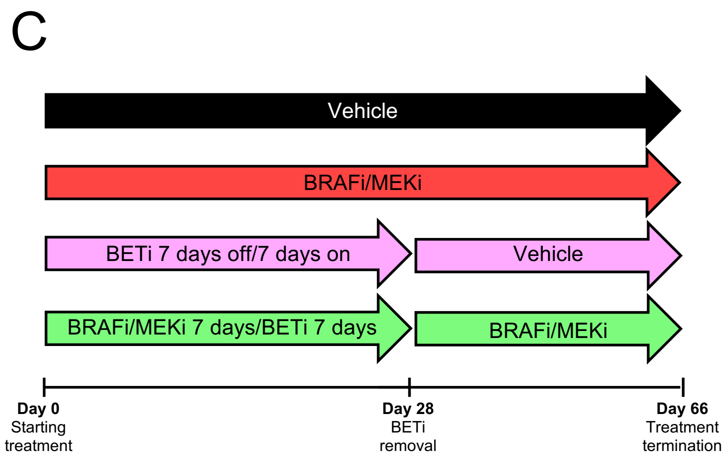

D

	CR after treatment termination	Drug toxicity symptoms followed by death
Vehicle	0/5 (0%)	0/5 (0%)
BRAFi/MEKi	0/5 (0%)	1/5 (20%)
BETi 7 days off/7 days on	0/5 (0%)	1/5 (20%)
BRAFi/MEKi 7 days/BETi 7 days	0/5 (0%)	1/5 (20%)

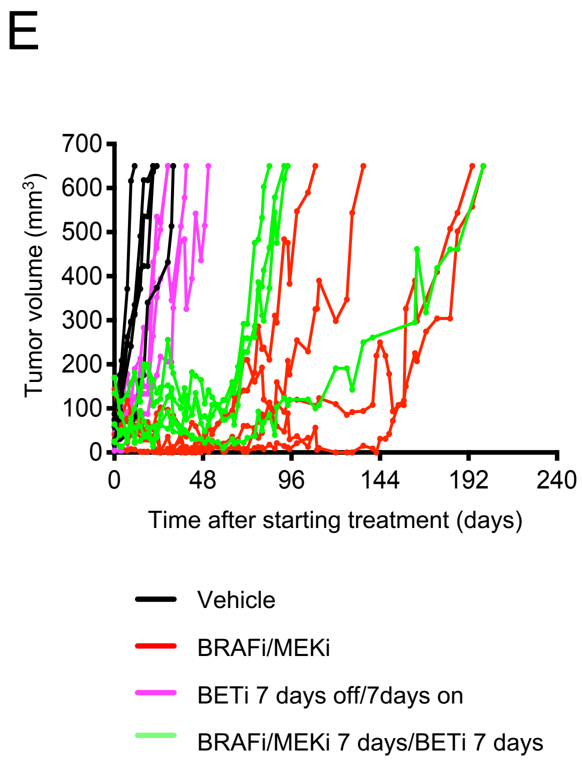

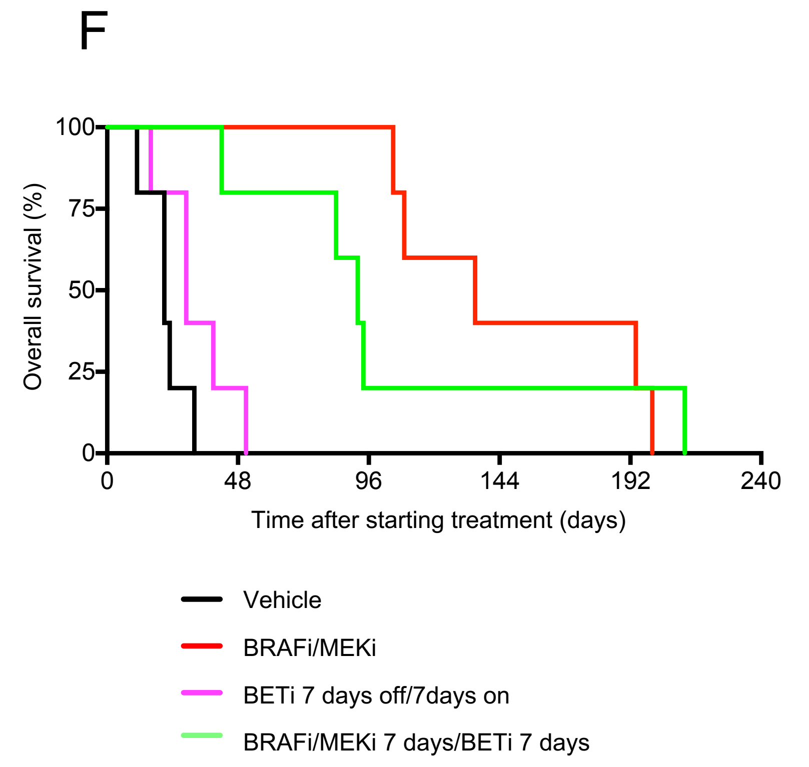

A

	CR after treatment termination	Drug toxicity symptoms followed by death
Vehicle	0/6 (0%)	0/6 (0%)
BRAFi/MEKi	0/6 (0%)	0/6 (0%)
2+5BETi	0/6 (0%)	0/6 (0%)
BRAFi/MEKi/2+5BETi	2/6 (33.4%)	1/6 (16.7%)

B

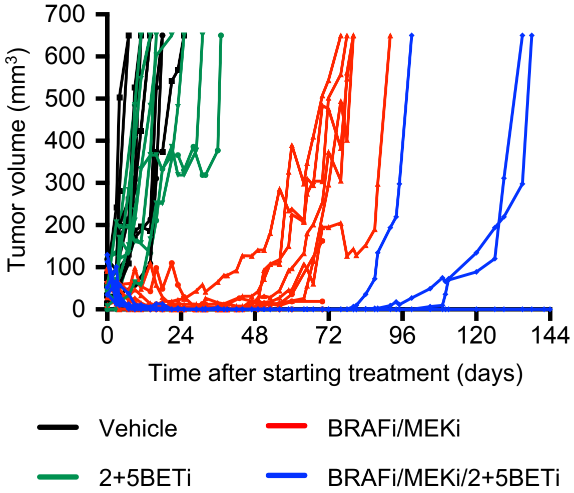

C

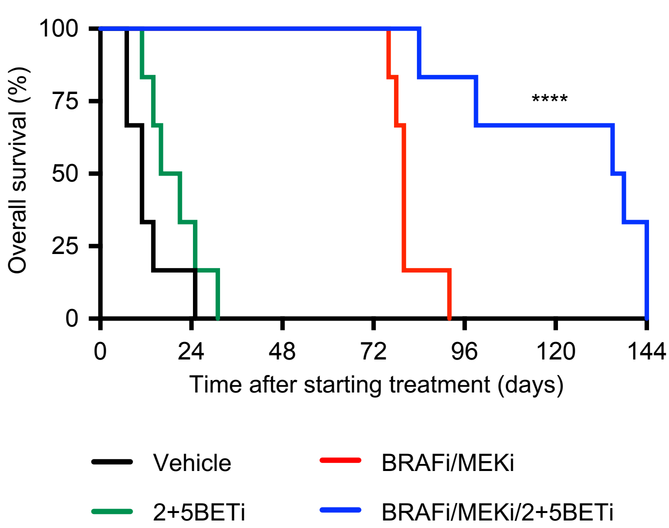

D

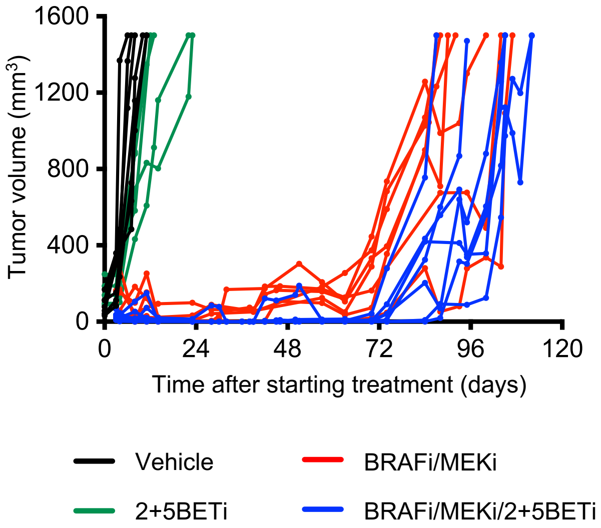

SUPPLEMENTARY FIGURE LEGENDS

Supplementary Figure 1. BET inhibition represses RTK gene expression following BRAF/MEK inhibitors treatment in BRAF-mutant melanoma. Relative gene expression of ERBB3 and PDGFRB for melanoma cell lines, A375, 1205Lu and M238, by qPCR after 24 hours of treatment combination using BRAFi/MEKi (50 nM dabrafenib/5 nM trametinib) plus BETi, either JQ1, 1 μM or PLX51107, 2 μM. Relative gene expression ratio for RTKs was compared to gene housekeeping expression, GAPDH, and normalized with BRAFi/MEKi for each respective cell line. *$p<0.05$, **$p<0.005$, ***$p<0.001$.

Supplementary Figure 2. Structures for inhibitors used in this manuscript. Source: https://pubchem.ncbi.nlm.nih.gov/

Supplementary Figure 3. Correlation between BRD2/4 levels and RTKs, ERBB3 and PDGFRB. (A) Scatter plots with trend lines of BRD4 mRNA versus ERBB3 mRNA and protein z-score data from BRAF-mutant TCGA cutaneous melanoma samples (n=142). **(B)** Scatter plots with trend lines of BRD2 mRNA versus PDGFRB mRNA z-score data from BRAF-mutant TCGA cutaneous melanoma samples (n=142). **(C)** Scatter plots with trend lines of BRD4 mRNA versus PDGFRB mRNA and protein z-score data from BRAF-mutant TCGA cutaneous melanoma samples (n=142). Samples were stratified into groups based on median expression levels. Survival analysis was performed using the Log-rank method via the survival (v 2.41.3) and survminer (v0.4.2) packages. **(D)** Number of patients harboring different BRAF mutations. **(E)** Spearman's correlation results for BRD2 and BRD4 versus ERBB3 using data from BRAFp.V600E/M/G-mutant patients.

Supplementary Figure 4. Correlation between BRD3 levels and RTKs, ERBB3 and PDGFRB. (A) Scatter plots with trend lines of BRD3 mRNA versus ERBB3 and PDGFRB mRNA z-score data from BRAF-mutant TCGA cutaneous melanoma samples (n=142). **(B)** Scatter plots with trend lines of BRD3 mRNA versus ERBB3 protein z-score data from BRAF-mutant TCGA cutaneous melanoma samples (n=142). **(C)** Scatter plots with trend lines of BRD3 mRNA versus phospho-ErbB3 protein z-score data from BRAF-mutant TCGA cutaneous melanoma samples (n=142). Samples were stratified into groups based on median expression levels. Survival analysis was performed using the Log-rank method via the survival (v 2.41.3) and survminer (v0.4.2) packages.

Supplementary Figure 5. BRD4 isoforms expression following BRD2 and BRD4 knockdown BRAF/MEK inhibitors treatment on BRAF-mutant melanoma. Western blot for BRD4 after BRD2 and BRD4 knockdown and 24 hours of treatment using BRAFi/MEKi (dabrafenib, 50 nM/trametinib, 5 nM) for the BRAF-mutant human melanoma cell lines, 1205Lu and M238. The siRNA scramble control (siCTRL), BETi (PLX51107, 2 μM) single agent and BRAFi/MEKi/BETi treatments for 24 hours were used as controls. Isoforms for BRD4 were identified as short (BRD4 S) and long (BRD4 L). Antibody used to detect BRD4 isoforms: Abcam #ab128874.

Supplementary Figure 6. Continuous BRAFi/MEKi/BETi in BRAF-mutant xenografts. (A) Treatment schema for vehicle (control), BRAFi/MEKi, BETi and BRAFi/MEKi/BETi chow continuous treatments for mice bearing 1205Lu xenografts. Sixty-six days after starting treatment schedule, remaining animals of all groups were removed from drug chow and switched to control chow to evaluate residual disease. All cohorts had 6 animals per group. **(B)** Percentage of animals which presented complete response (CR) after cessation of therapy or symptoms of drug toxicity followed by death during treatment. **(C)** Animal weight during drug treatments. **(D)** Tumor growth data for mice bearing 1205Lu xenografts on treatment doses described above. **(E)** Cell

proliferation curves by IncuCyte assay over 3 weeks of different treatment schedules using BRAFi/MEKi plus BETi (PLX51107, 2μM) for 1205Lu cells. The schedule treatments of 4 days on/3 days off BETi (4+3BETi) combined with continuous BRAFi/MEKi (BRAFi/MEKi/4+3BETi)**;** 2 days on/5 days off BETi (2+5BETi) combined with continuous BRAFi/MEKi (BRAFi/MEKi/2+5BETi), 7 days off/7 days on BETi alone; 7 days on/7 days off BRAFi/MEKi; and 7 days of BRAFi/MEKi followed by 5 days of BETi were compared to vehicle (DMSO), BRAFi/MEKi, BETi alone and BRAFi/MEKi/BETi continuous treatments.

Supplementary Figure 7. Intermittent BETi treatment with intermittent and continuous BRAFi/MEKi in 1205Lu xenografts. (A) Tumor growth for Figures 6C-D scheduling groups during drug treatment. **(B)** Animal weight loss for Figures 6C-D scheduling groups during drug treatment. **(C)** Treatment schedule schema for intermittent treatment of 7 days off/7 days on BETi alone and 7 days of BRAFi/MEKi followed by 7 days of BETi alone in a 14-day cycles; compared to vehicle (control) and BRAFi/MEKi continuous chow treatments; compared to vehicle (control) alone or BRAFi/MEKi alone continuous chow treatments. After that, BETi was removed and remaining animals were maintained either in vehicle or BRAFi/MEKi chow. On day 66, remaining mice bearing 1205Lu xenografts were switched to control chow to evaluate residual disease. All cohorts had 5 animals per group. **(D)** Percentage of animals which presented complete response (CR) after cessation of therapy or symptoms of drug toxicity during treatment. **(E)** Tumor growth for 1205Lu xenografts on treatment doses described above. **(F)** Animal overall survival (criteria: death or tumor size reaches 650 mm^3).

Supplementary Figure 8. Intermittent BETi treatment with continuous BRAFi/MEKi in A375 cells and PDX xenografts. (A) Treatment of mice bearing A375 xenografts with intermittent treatment of 2 days on/5 days off BETi chow (2+5BETi) combined with continuous BRAFi/MEKi (BRAFi/MEKi/2+5BETi) chow, in a 7-day cycles by day 28; compared to vehicle (control) alone or

BRAFi/MEKi alone continuous chow treatments. Percentage of animals which presented complete response (CR) after cessation of therapy or symptoms of drug toxicity. After day 24, BETi was removed and remaining animals were maintained either in vehicle or BRAFi/MEKi chow. On day 66, remaining mice bearing A375 xenografts were switched to control chow to evaluate residual disease. All cohorts had 6 animals per group. **(B)** Tumor growth data for mice bearing A375 xenografts on treatment doses described above. **(C)** Animal survival (criteria: death or tumor size reaches 650 mm^3). Kaplan-Meier analysis (****p<0.0001). **(D)** Tumor growth for Figures 6E-F scheduling groups during drug treatment.
